# NMDAR modulators as rapid antidepressants: Converging and distinct signaling mechanisms

**DOI:** 10.15761/icm.1000173

**Published:** 2020-02-04

**Authors:** Boyoung Lee, Santosh Pothula, Ronald S Duman

**Affiliations:** Department of Psychiatry, Yale University School of Medicine, New Haven, CT, USA

**Keywords:** NMDA receptor modulator, Ketamine, Rapastinel, depression

## Abstract

Dysregulation of the glutamatergic system underlies the pathophysiology of depression. Both negative and positive modulation of NMDARs exert rapid and sustained antidepressant effects by reversing the dysregulated glutamatergic system. Research in the past decades has identified key signaling pathways activated by these rapid acting antidepressants. Here, we review the converging signaling mechanisms shared by rapid acting antidepressants and discuss the recent progress on distinct actions of NMDAR antagonists and NMDAR positive modulators to trigger rapid antidepressant actions.

## Introduction

Major depressive disorder (MDD) is a debilitating psychiatric disorder and one of the leading causes of disability worldwide. Although the monoaminergic hypothesis of depression dominated the field for several decades, research in the past two decades demonstrates that the glutamatergic system plays a central role in the pathophysiology of MDD [1]. Conventional antidepressants targeting monoaminergic systems have limitations of time lag for therapeutic effect and low efficacy. In contrast, low subanesthetic doses of the N-methyl-D-aspartate (NMDA) receptor antagonist ketamine exerts rapid and sustained antidepressant effects in both clinical and preclinical studies [2,3]. However, not all NMDAR antagonists exert these effects consistently in both clinical and preclinical studies [4,5]. Recently, the FDA approved the (S) enantiomer of ketamine, esketamine in the form of a nasal application referred to as Spravato for the treatment of MDD, but esketamine, like racemic ketamine is also associated with psychotomimetic and dissociative side effects. Recently, an NMDA receptor positive allosteric modulator (NMDAR-PAM), rapastinel has also received attention as a rapid acting antidepressant, in particular it lacks the side effects of ketamine. This raises a question of how both an NMDAR antagonist and PAM with opposing actions on NMDARs exert similar rapid and sustained antidepressant effects. Despite the failure of rapastinel in phase 3 clinical trials, it will be interesting to understand the key mechanisms underlying the actions of these NMDAR PAMs considering the fact that high placebo response is associated with intravenous administration of rapastinel in clinical trials and currently orally administered NMDAR PAMs like AGN-241751 are in phase II clinical trials [4].

Here, we review previous and current research on the mechanism underlying the actions of rapastinel and compare with ketamine, including both unique convergent mechanisms underlying the actions of these rapid antidepressants. We discuss novel NMDAR-PAMs and their therapeutic potential for treatment of depression and neuropsychiatric disorders.

## Converging and distinct signaling mechanisms for rapid acting antidepressants

Rapastinel is a threonine-proline-proline-threonine (TPPT) tetrapeptide derived from a hypervariable region cloned and sequenced from the monoclonal antibody B6B216 [6]. Rapastinel was initially suggested to act as a NMDAR glycine site partial agonist but recent studies have shown that it enhances NMDAR activity by its actions at an allosteric site. Similar to ketamine, the efficacy of rapastinel as a rapid and long-lasting antidepressant has been shown in many rodent depression models [8,9] ongoing studies in our lab; and in humans [10]. Importantly, as mentioned above, rapastinel has shown no clinically significant dissociative or psychotomimetic side effects like ketamine [8].

Recently, our studies focused on the converging cellular and molecular mechanisms in the antidepressant actions of rapastinel, an NMDAR-PAM as well as the NMDAR antagonist, ketamine. Our previous studies reported that activation of the mTORC1 signaling pathway (local protein synthesis), through ERK/AKT activation acts as a key underlying convergent mechanism for the fast-acting antidepressant properties of ketamine [11]. In addition, BDNF release and TrkB receptor activation by ketamine has been suggested to lead the activation of ERK and mTORC1 signaling [12,13]. Interestingly, Lepack et al. [14] demonstrated that the antidepressant action of rapastinel requires activation of similar pathways. Rat primary cortical neuronal cultures were used in this study to examine and compare the cellular and molecular mechanisms underlying the actions of ketamine and rapastinel. The results demonstrated that low concentrations of ketamine or rapastinel rapidly increased the levels of the phosphorylated and activated forms of ERK (pERK) and a downstream target of mTORC1, p70S6 kinase (pS6K), in a concentration and time dependent manner. In addition, both drugs rapidly increased BDNF release into the culture media. This study also demonstrated that induction of BDNF release, as well as activation of pERK is blocked by incubation with an AMPA receptor antagonist, NBQX. The requirement of BDNF release and activation of TrkB-RhoGTPase for antidepressant effects of rapastinel was also confirmed in animal model of depression [15]. Lepack et al. [14] further tested whether the blockade of BDNF release by NBQX is activity dependent. To that end, a neuronal silencing agent, muscimol (GABA_A_ receptor agonist) was used to inhibit glutamatergic neuronal activation; the results demonstrated that muscimol completely blocked the increase in phospho-ERK and BDNF release induced by either ketamine or rapastinel. Together, these findings indicate that two different rapid acting antidepressants with opposing NMDAR effects share common molecular and cellular mechanisms.

Liu et al. [16] further characterized the underlying mechanisms of rapastinel *in vivo* to reveal mechanistic differences between ketamine and rapastinel. This study demonstrated that like ketamine, a single dose of rapastinel rapidly activated mTORC1 pathway in medial prefrontal cortex (mPFC). Infusion of rapamycin, the selective mTORC1 inhibitor, blocked the antidepressant-like behavioral actions of rapastinel, demonstrating a requirement for the mTORC1 pathway. Further, rapastinel increased the number and function of spine synapses in the apical dendritic tuft of layer V pyramidal neurons in the mPFC, an effect also reported after ketamine treatment [11]. Therefore, even *in vivo*, this study identified similar molecular and cellular mechanisms for ketamine and rapastinel. However, in this study, the authors also reported some differences. Rapastinel is a centrally active, intravenously administered (non-orally active) peptide, so Liu et al. [16] injected rapastinel and ketamine intravenously to compare the effects on mTORC1 signaling. Both pS6K and p4EBP1 were increased significantly by either ketamine or rapastinel. However, pmTOR, pERK and pAKT were not significantly increased by ketamine. Instead, intraperitoneal injection of ketamine increased pmTOR, pERK and pAKT along with pS6K and p4EBP1. The authors proposed that intravenous injection accelerated the action of ketamine thus facilitating the time to rapidly return to basal levels for those proteins. Thus, rapastinel might have a longer-lasting effect time on phosphorylation of mTOR, ERK and AKT than ketamine when injected intravenously. These results suggest that ketamine and rapastinel may have different reaction times at the NMDA receptor or different pharmacokinetic profiles.

It is also likely that ketamine and rapastinel could act at NMDARs on different cell types or recruit unknown distinct signaling pathways that converge on ERK or mTOR signaling. Electrophysiological studies also demonstrated that rapastinel significantly increased the frequency of EPSCs to hypocretin, a measure of thalamocortical synapses. However, rapastinel did not increase the frequency of EPSCs to 5-HT, a measure of cortical-cortical response mediated by the 5-HT2A receptor. Previous studies revealed that ketamine increases both hypocretin- and 5-HT induced ESPC frequency [11,12,17,18]. Therefore, the authors suggested that the differences in the 5-HT2A receptor synaptic and behavioral responses between ketamine and rapastinel may be related to the lack of ketamine-like psychotomimetic side effects for rapastinel. Altogether, this study clearly showed that there are differences in the cellular or molecular actions of ketamine and rapastinel even though both agents require activation of converging signaling pathways and changes in synaptic plasticity.

## Initial cellular trigger for rapid antidepressants: NMDAR antagonism vs. positive modulation

While both rapastinel and ketamine physically target NMDA receptors and share converging molecular signaling mechanisms, the two molecules have opposing actions on NMDARs. Ketamine is a non-competitive NMDAR antagonist while rapastinel acts as an NMDAR-PAM but how both these drugs exert similar rapid and sustained antidepressant effects and activate convergent downstream signaling pathways has been an unanswered question. One of the most accepted hypotheses that addresses this question is that ketamine and rapastinel could modulate NMDARs on different cell types in mPFC. Recent studies from our lab addressed this issue by using a Crerecombinase dependent viral shRNA approach to produce cell type specific knockdown of the GluN2B subunit of NMDARs in different Cre-lines (Camk2a-, Gad1-, Sst- and Pval-Cre mice) [19]. GluN2B was targeted based on evidence that it is a key NMDAR subunit that has been implicated in actions of ketamine [20,21]. The study found that GluN2B knockdown in GABA (Gad1) interneurons, as well as GABA subtypes expressing somatostatin (Sst) or parvalbumin (Pvalb) blocked the antidepressant actions of ketamine but not in glutamatergic (Camk2a) neurons. Therefore, the study demonstrated that GluN2B-NMDARs on GABAergic inhibitory neurons are the initial cellular trigger for the actions of ketamine. In addition, electrophysiology data revealed a decrease in sIPSCs and an increase in sEPSCs in mPFC by ketamine both in male and female mice supporting the hypothesis that ketamine blocks NMDARs on tonic firing GABAergic interneurons, in turn decreasing inhibitory inputs onto excitatory pyramidal neurons (disinhibition hypothesis). Ongoing studies are evaluating the role of the GluN2B on excitatory vs. GABAergic inhibitory neurons in the actions of rapastinel to tease apart the distinct and unique cellular actions of NMDAR PAMs vs antagonists that trigger the activation of converging signaling pathways and drive rapid and sustained antidepressant actions.

## Conclusion

**Figure 1.**
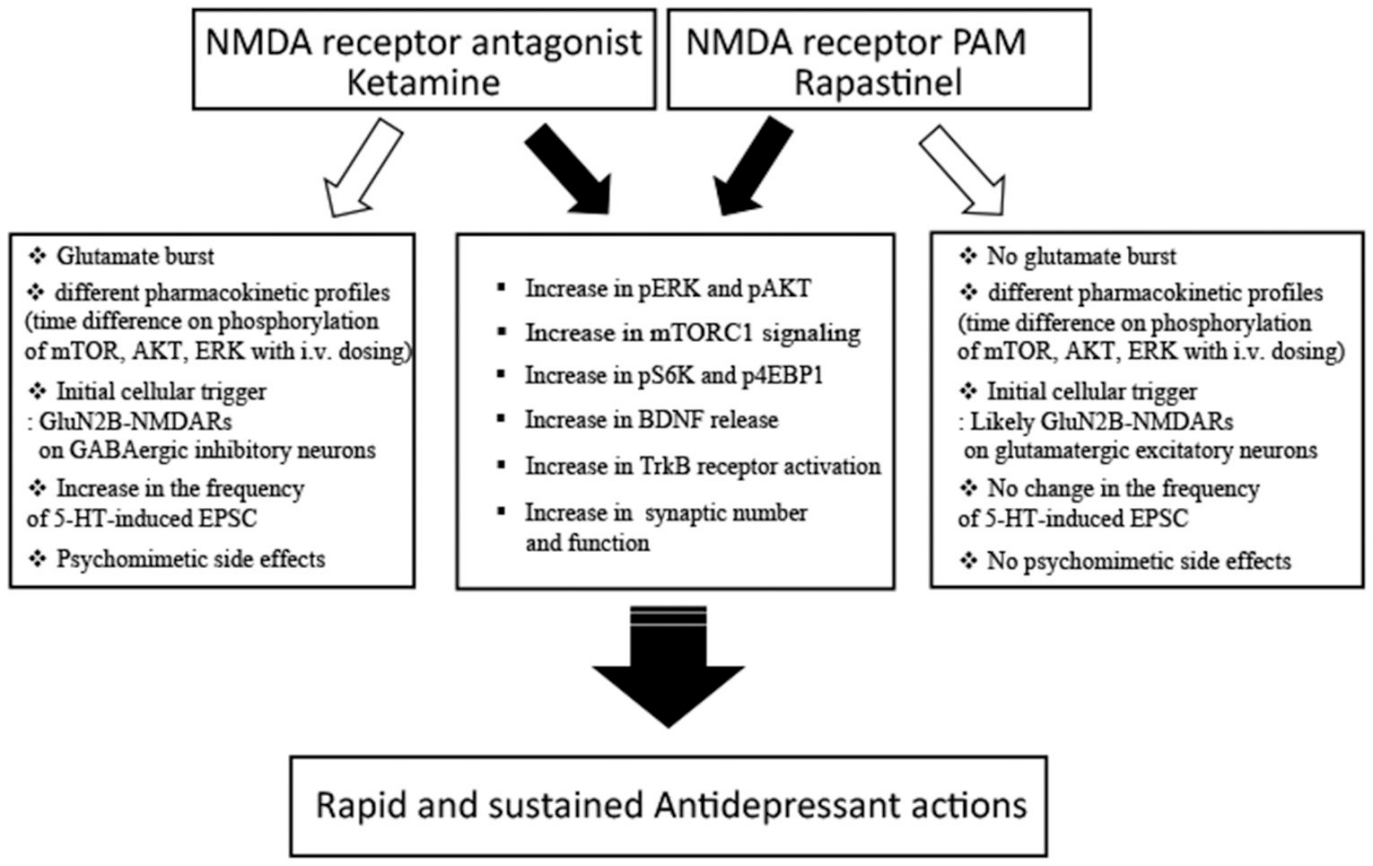
Summary of conversing and distinct signaling mechanisms underlying the rapid and sustained antidepressant actions of ketamine and rapastinel.

Ketamine and rapastinel produce rapid and sustained antidepressant effects. Both molecules increase mTORC1, pERK and pAKT, and BDNF release, resulting in increased synaptic number and function that are associated with antidepressant responses (Figure 1). However, there are clear differences: one is an NMDAR antagonist and the other is NMDAR-PAM. Ketamine increases both hypocretin- and 5-HT induced ESPC frequency [11,12,17,18] but rapastinel only increases the frequency of hypocretin-induced EPSCs not the frequency of 5-HT-induced EPSCs (Figure 1). Ketamine acts on GluN2B containing NMDAR on GABAergic interneurons; ongoing studies are testing the initial target for rapastinel, which could act directly on GluN2B containing NMDARs on glutamatergic neurons (Figure 1). Esketamine has received FDA-approval but rapastinel has failed in recent phase 3 clinical trials. However, Kato et al. [15] addressed several issues for the failure of rapastinel in clinical trials. Nevertheless, it is important to understand the distinct and converging actions of NMDAR antagonists and NMDAR-PAMs to identify novel targets for the development of safer and efficacious rapid antidepressant. Recently, novel NMDAR-PAMs have been developed which are orally active and are currently being examined for their therapeutic efficacy in depression and other psychiatric disorders, including like post-traumatic stress disorder (PTSD). We are currently testing a novel NMDAR-PAM in animal models of PTSD and have found promising results (unpublished data). Therefore, NMDAR-PAMs could still be potential novel therapeutic options for the treatment of depression and other psychiatric disorders.
